# Role of FGF21 in mediating the effect of phosphatidylcholine on GBM

**DOI:** 10.3389/fonc.2024.1428025

**Published:** 2024-09-02

**Authors:** Peng Wang, Xin Zhang, Boan Xiao, Jiecai Ouyang, Jingjing Zhang, Xiaobin Peng

**Affiliations:** The Fifth Affiliated Hospital, Southern Medical University, Guangzhou, China

**Keywords:** PC16, FGF21, Mendelian randomization, mediation, GBM

## Abstract

**Objective:**

The causal relationship and mechanisms between lipids and glioblastoma (GBM) remain unclear. This study aims to investigate the independent causal relationship between liposomal phosphatidylcholine 16:0_22:6 (PC16) and GBM, and to identify the potential mediating role of the inflammatory factor-fibroblast growth factor 21(FGF21).

**Methods:**

Utilizing summary statistics from genome-wide association studies (GWAS) of lipids (179 types in 7174 Finnish individuals), GBM (243 cases and 287137 controls), and inflammatory factors (91 types in 14824 European individuals), a two-sample Mendelian Randomization (MR) approach was employed to establish the causal link between liposomal PC16 and GBM. Additionally, a two-step MR method was used to quantify the proportion of the causal effect of PC16 on GBM that is mediated by the inflammatory factor FGF21.

**Results:**

MR analyses revealed a strong causal relationship between PC16 and GBM (OR=1.72, 95% CI: 1.11-2.68, P=0.016), but no reverse causality was observed from GBM to PC16 (OR=1.01, 95% CI: 0.99-1.02, P=0.38). Mediation analysis showed a strong causal relationship between PC16 and the FGF21 (OR = 0.94, 95% CI: 0.89-0.99, P=0.018) as well as between FGF21 and GBM (OR = 0.42, 95% CI: 0.25-0.71, P=0.001), with the mediation effect accounting for 9.78% of the total effect. This suggests that the causal relationship between PC16 and GBM is likely mediated by the intermediary factor FGF21. No evidence of pleiotropy was found in the sensitivity analysis of these positive results.

**Conclusion:**

In summary, the findings of this study suggest that liposomal PC16 may increase the risk of GBM occurrence, and FGF21 may play a significant mediating role in this causal relationship.

## Introduction

Brain tumors are among the most difficult malignant tumors to cure, with gliomas being one of the most biologically invasive, complex, and heterogeneous brain tumors in the central nervous system of adults (1, 2). Glioblastoma (GBM), a World Health Organization (WHO) grade IV glioma, accounts for 12-15% of all brain tumors. Patients have a median survival of only 14-16 months, with a 5-year survival rate of approximately 5% (3–5). GBM exhibits high malignancy with low cure rates and high recurrence rates. The main clinical treatments are surgery, radiotherapy, and chemotherapy. However, due to its complexity, variable biological characteristics, and limited understanding of the developmental causes of glioblastoma, the clinical prognosis is very poor (6). Therefore, further exploration of the pathogenesis of GBM, identification of its endogenous biomarkers, determination of potential targets, and development of effective treatment methods are of significant importance for improving patient survival rates.

Phosphatidylcholine and its derivatives, a class of phospholipids, are one of the main components of cellular biological membranes and help maintain cell structure and function (7). They are also sources of lipid second messengers and determinants of the cell cycle process. The choline metabolites produced by their synthesis and catabolism contribute to proliferative growth and programmed cell death (8). Lipids have a variety of biological functions, reflecting the physiological and pathological states of cells, tissues, and organs. Alterations in lipid metabolism are among the most significant metabolic changes in the development of cancer. Under the influence of carcinogenic and environmental factors, cancer cells widely modify their metabolism to survive and develop in the constantly changing microenvironment (9). Studies have shown that total choline metabolites, including choline, phosphocholine, and phosphatidylcholine, are elevated in various tumors and cancer cells (10, 11). Therefore, this study aims to explore the potential association between phosphatidylcholine expression levels and the risk of GBM.

Fibroblast growth factor 21(FGF21), an intermediary factor, is a novel regulator of glucose and lipid metabolism produced by the liver or other tissues, skeletal muscle, and pancreatic beta cells. It plays multiple roles in development, organogenesis, and metabolism (12). For example, the FGF21/FGFR signal promotes neurite outgrowth in an L1-dependent manner and plays an important role in neural development (13). FGF21 has potent protective properties in neurons, such as inhibiting glutamate-induced death of primary rat brain neurons and D-galactose-induced brain aging in mice (14, 15). This suggests that FGF21 not only affects peripheral system glucose and lipid metabolism but also has significant neurobiological consequences in brain cells, including neurons and glial cells. This also hints that FGF21 might be an intermediary factor affecting the causal relationship between PC16 and GBM. Therefore, this study uses Mendelian Randomization (MR) to analyze the causal relationship between PC16 and GBM and to verify whether FGF21 mediates the relationship between PC16 and GBM.

MR studies are a method of causal inference that uses single nucleotide polymorphisms (SNPs) associated with exposure as instrumental variables (IVs) to infer causal associations between exposure and outcome (16). MR leverages Mendel’s laws of genetics, as genetic variants are randomly distributed during gamete formation and are mostly unaffected by environmental or lifestyle factors, thus reducing bias from unmeasured confounders or confounding factors while avoiding reverse causality (17). Therefore, our goal is to determine through MR analysis whether PC16 has a causal relationship with GBM and to clarify whether FGF21 as an intermediary participates in mediating this causal relationship and to quantify its mediation proportion.

## Materials and methods

### Research design

**Figure 1:**
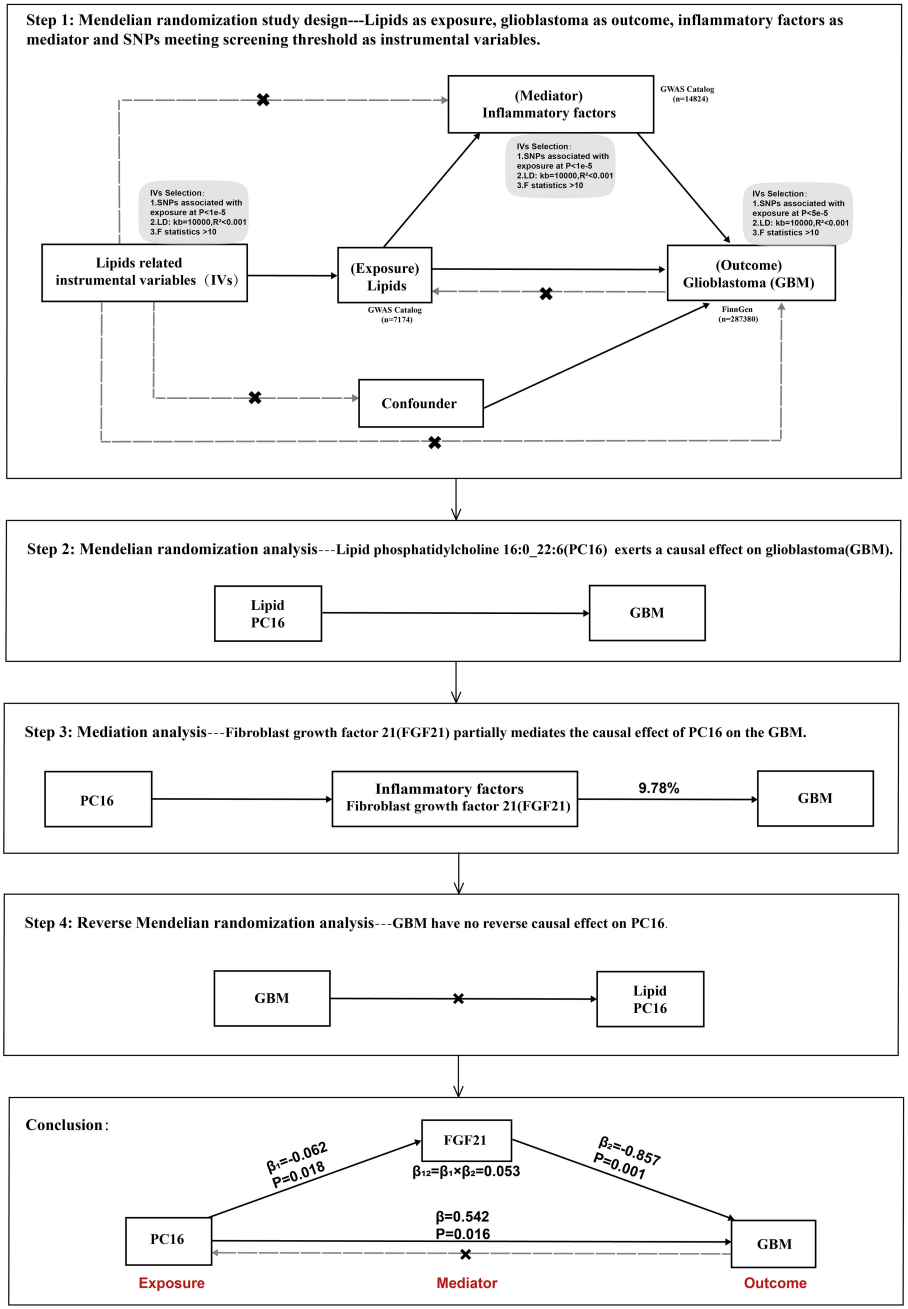
Overview of this Mendelian randomization (MR) analysis, including study design and main findings.

This study utilizes publicly available genome-wide association study (GWAS) summary statistics. Phosphatidylcholine 16:0 (PC16) was considered the exposure factor, glioblastoma multiforme (GBM) as the outcome, and fibroblast growth factor 21 (FGF21) was included for mediation analysis. Initially, the total causal effect of PC16 on GBM was analyzed, followed by the causal relationship between PC16 and FGF21. Subsequent causal inference was made between FGF21 and GBM, and finally, the proportion of the effect mediated by the intermediary factor in the causal relationship between PC16 and GBM was assessed (**Figure 1**).

### Data sources

The Mendelian randomization (MR) study utilized data from large-scale GWAS. The lipidomic data were derived from a genome-wide analysis of 179 lipid species in 7174 Finnish individuals by Ottensmann et al. (accession numbers: GCST90277238-GCST90277416) (18). Data on inflammation markers came from a genetic study on circulating inflammatory proteins by Zhao, J.H et al., including 14824 participants of European descent (accession numbers: GCST90274758 - GCST90274848) (19). GBM data were obtained from the FinnGen database, which included 243 cases and 287137 controls, all of European descent (20). Each study had corresponding ethical review approvals and patient informed consent, eliminating the need for additional ethical approval.

### Instrumental variable selection

MR studies require that the instrumental variables (IV) satisfy three assumptions: (1) IV is significantly associated with the exposure; (2) IV is not associated with any confounders; (3) IV influences the outcome only through the exposure. To meet these criteria, SNPs that reached genome-wide significance thresholds were extracted as IVs. For lipidomic PC16 and inflammatory marker FGF21, we selected SNPs with p < 1e-5, and for GBM, SNPs with p < 5e-5. We retained SNPs without linkage disequilibrium (LD) (kb=10000, R^2^ <0.001) to ensure the SNPs were not closely related. SNPs significantly associated with the outcome at the genome-wide level (P<5e-8) were excluded. Variance (R^2^) and F-statistics were used to estimate the association strength between the selected IVs and the exposure, with weak instruments (F-statistic <10) being removed to avoid bias. SNPs with allele frequencies inconsistent between exposure and outcome, as well as palindromic SNPs with intermediate allele frequencies, were excluded. The rigorously filtered SNPs were used for the final causal analysis.

### MR analysis

The main method used for this analysis was the inverse-variance weighted (IVW) method. IVW is powerful for causal detection but requires that genetic variants impact the outcome only through the exposure in the study. Despite attempts to exclude known confounder-related SNPs, many unknown confounders could still introduce bias. Alternative methods, including MR-Egger, weighted median, and Bayesian MR (BWMR), were also utilized to assess the robustness of the results. However, if the results among the different methods were inconsistent, IVW was prioritized as the primary method for estimating causal effects, given its common use in MR analysis and its robustness in the absence of directional pleiotropy (21).

### Sensitivity analysis

Cochran’s Q was used to assess heterogeneity among SNPs, indicating no heterogeneity when P>0.05. MR-Egger and MR-PRESSO were employed for pleiotropy testing. The “leave-one-out” approach was used to assess the influence of individual SNPs on the causal relationship. Sensitivity analysis was undertaken to verify the reliability of the results.

### Mediation analysis

The total effect of PC16 on GBM was decomposed into the direct effect of PC16 on GBM and the indirect effect mediated by the intermediary variable. A two-step MR design was further used for mediation analysis, exploring whether FGF21 mediated the causal pathway from PC16 to GBM. The percentage of the mediation effect was calculated by dividing the indirect effect by the total effect.

### Statistical analysis

The “Two-Sample MR” package was used for MR causal estimation analysis, and the “MR-PRESSO” package for MR pleiotropy and heterogeneity analysis. MR estimates were presented as odds ratios (OR) with 95% confidence intervals (CI). All statistical computations were performed using R version 4.2.3.

## Results

### Association of PC16 with GBM

**Figure 2:**
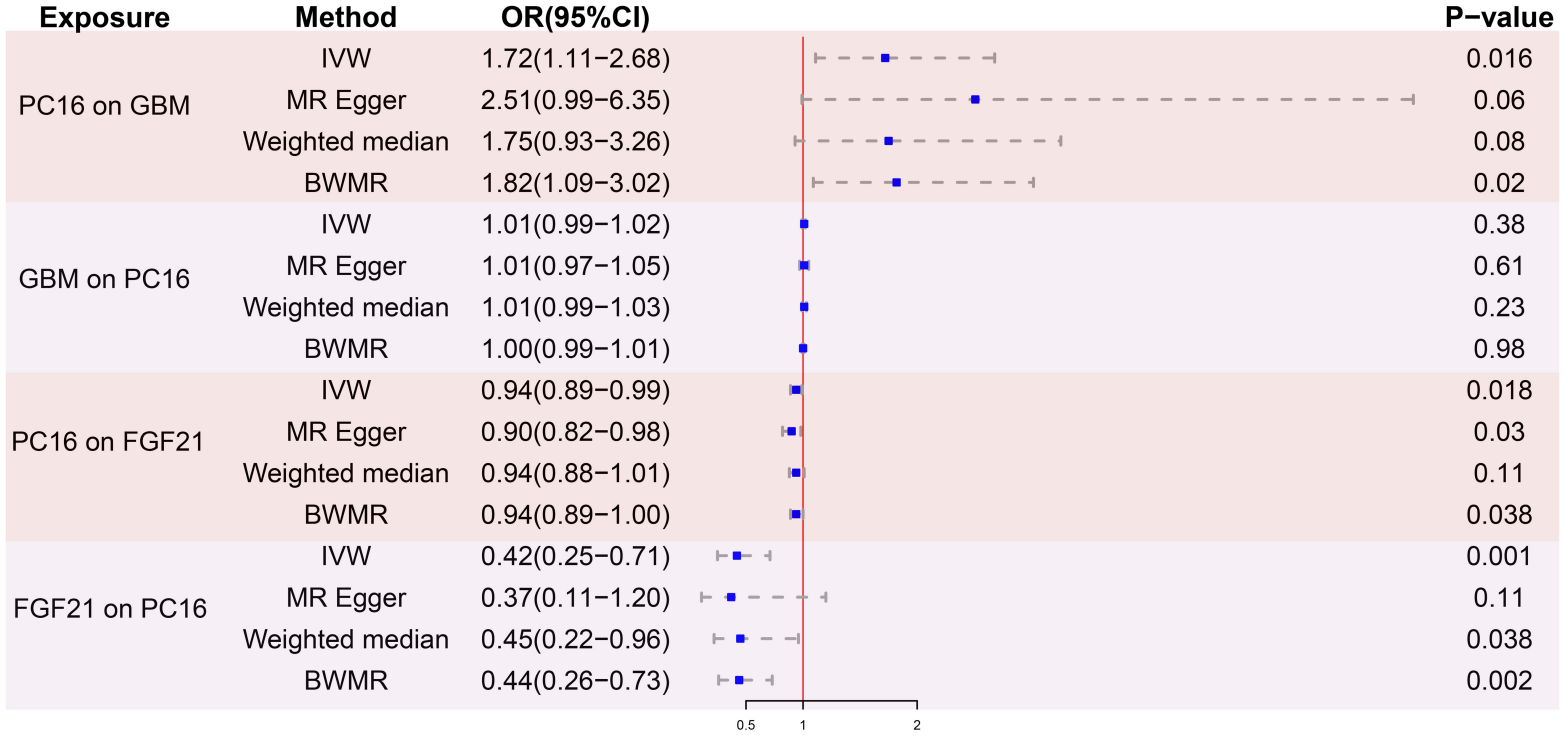
MR analysis results. PC16, phosphatidylcholine 16:0_22:6; GBM, glioblastoma; FGF21, fibroblast growth factor 21; IVW, inverse variance weighted; BWMR, bayesian weighted mendelian randomization; OR, odds ratio; CI, confidence interval; SNPs, single nucleotide polymorphisms.

**Figure 3:**
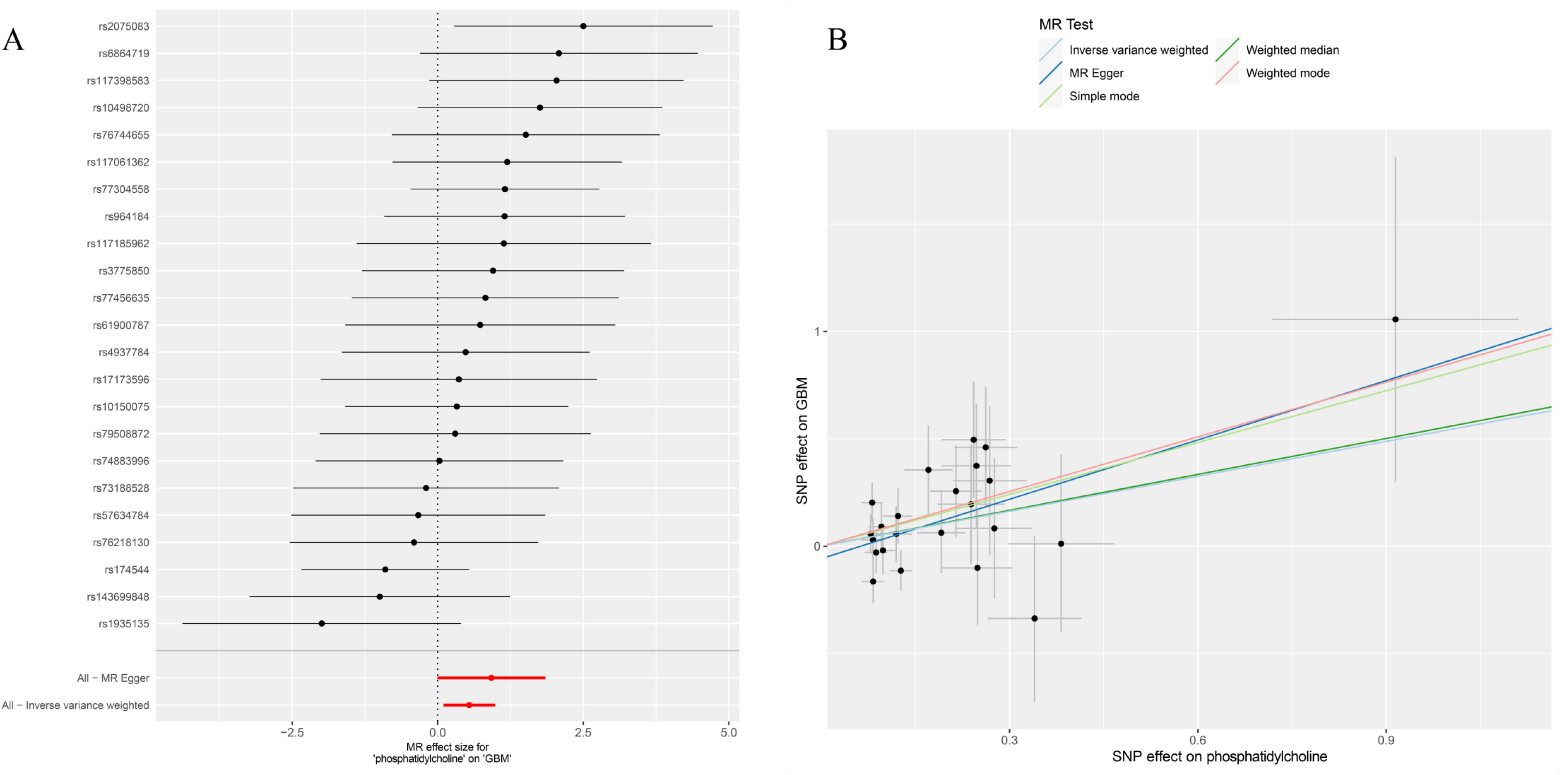
**(A)** Forest plot to visualize causal effect of each single SNP on total GBM risk; **(B)** Scatter plots to visualize causal relationship between FGF21 and GBM from different methods. SNP, single nucleotide polymorphism; MR, mendelian randomization; GBM, glioblastoma.

After removing palindromic and ambiguous SNPs, and those with linkage disequilibrium, a total of 23 SNPs were included in this study. The F-statistics of the PC16-exposed SNPs ranged from 19.8 to 53.9, all exceeding the threshold of 10, showing a strong intensity. This indicates no weak IVs bias, which means the results are reliable (**Supplementary Table S1**). The causal relationship between genetically predicted PC16 and GBM was estimated through regression methods such as Inverse Variance Weighting (IVW), MR-Egger, weighted median, and BWMR. Among all MR methods, the IVW method showed a significant positive correlation between genetically predicted PC16 expression and GBM (OR=1.72, 95% CI: 1.11-2.68, P= 0.016, **Figures 2**, **3**), and BWMR showed the same result (OR=1.82, 95% CI: 1.09-3.02, P= 0.02, **Figure 2**). However, after extracting the reliable genetic instrumental variables of GBM (**Supplementary Table S2**), a reverse MR analysis was conducted. The results showed that GBM has no reverse causal relationship with PC16 expression (IVW: OR=1.01, 95% CI: 0.99-1.02, P= 0.38; BWMR: OR=1.00, 95% CI: 0.99-1.01, P= 0.98, **Figure 2**).

### Association of PC16 with FGF21

**Figure 4:**
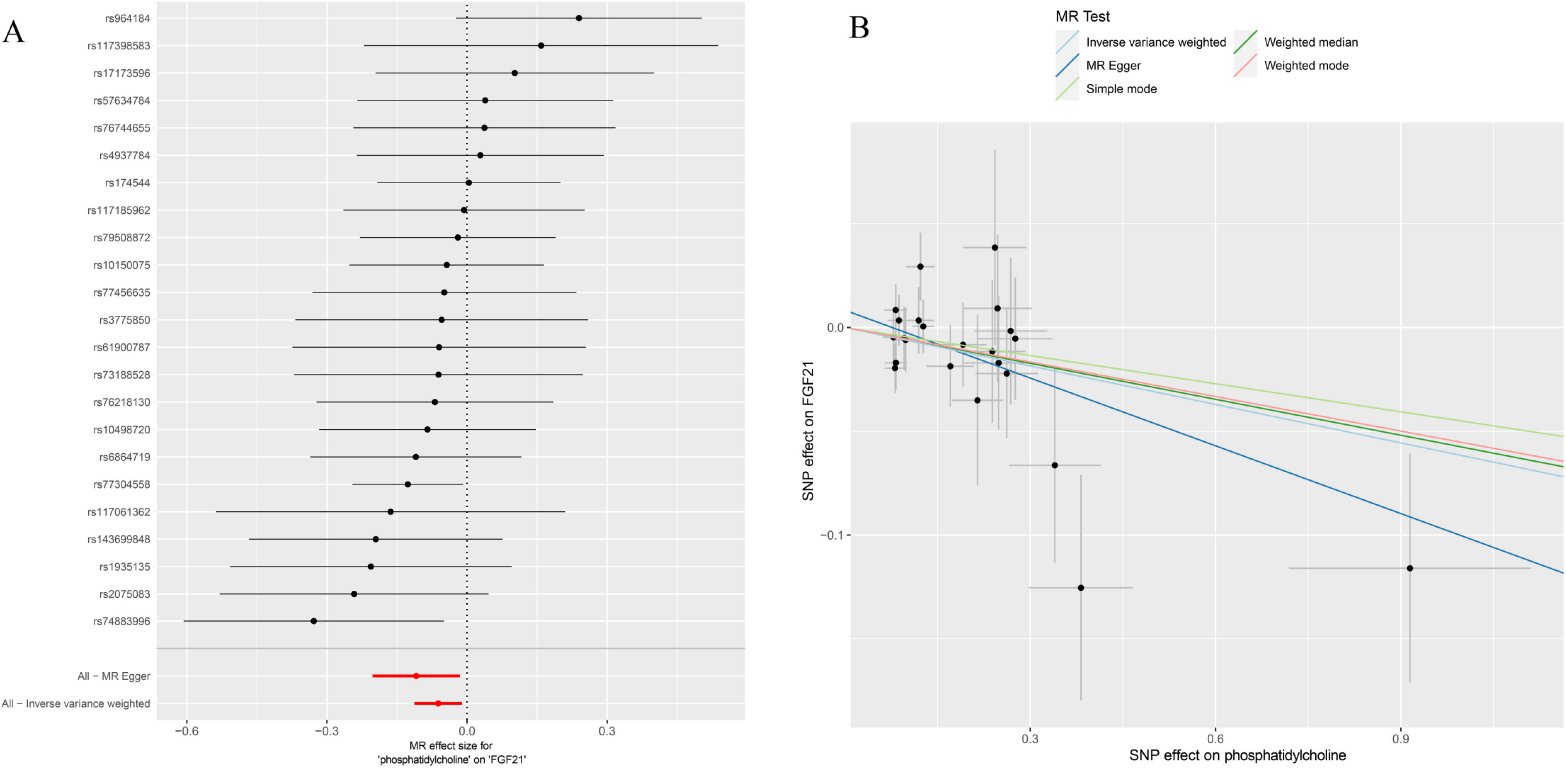
**(A)** Forest plot to visualize causal effect of each single SNP on FGF21; **(B)** the scatter plots to visualize causal relationship between PC16 and FGF21 from different methods. SNP, single nucleotide polymorphism; MR, mendelian randomization; PC16, phosphatidylcholine 16:0_22:6; FGF21, fibroblast growth factor 21.

After screening for strong instrumental variables and removing palindromic and ambiguous SNPs, we extracted 23 genome-wide significant SNPs as instrumental variables. The F-statistics of the genetic instrumental variables of PC16 ranged from 19.8 to 53.9 (**Supplementary Table S3**). Based on the IVW method, genetically predicted PC16 and FGF21 are negatively correlated (OR = 0.94, 95% CI:0.89-0.99, P=0.018) (**Figures 2**, **4A**). MR - Egger (OR =0.90, 95% CI:0.82-0.98, P=0.03) and BWMR (OR =0.94, 95% CI:0.89-1.00, P=0.038) yielded results consistent with the IVW method (**Figures 2**, **4B**).

### Association of FGF21 with GBM

**Figure 5:**
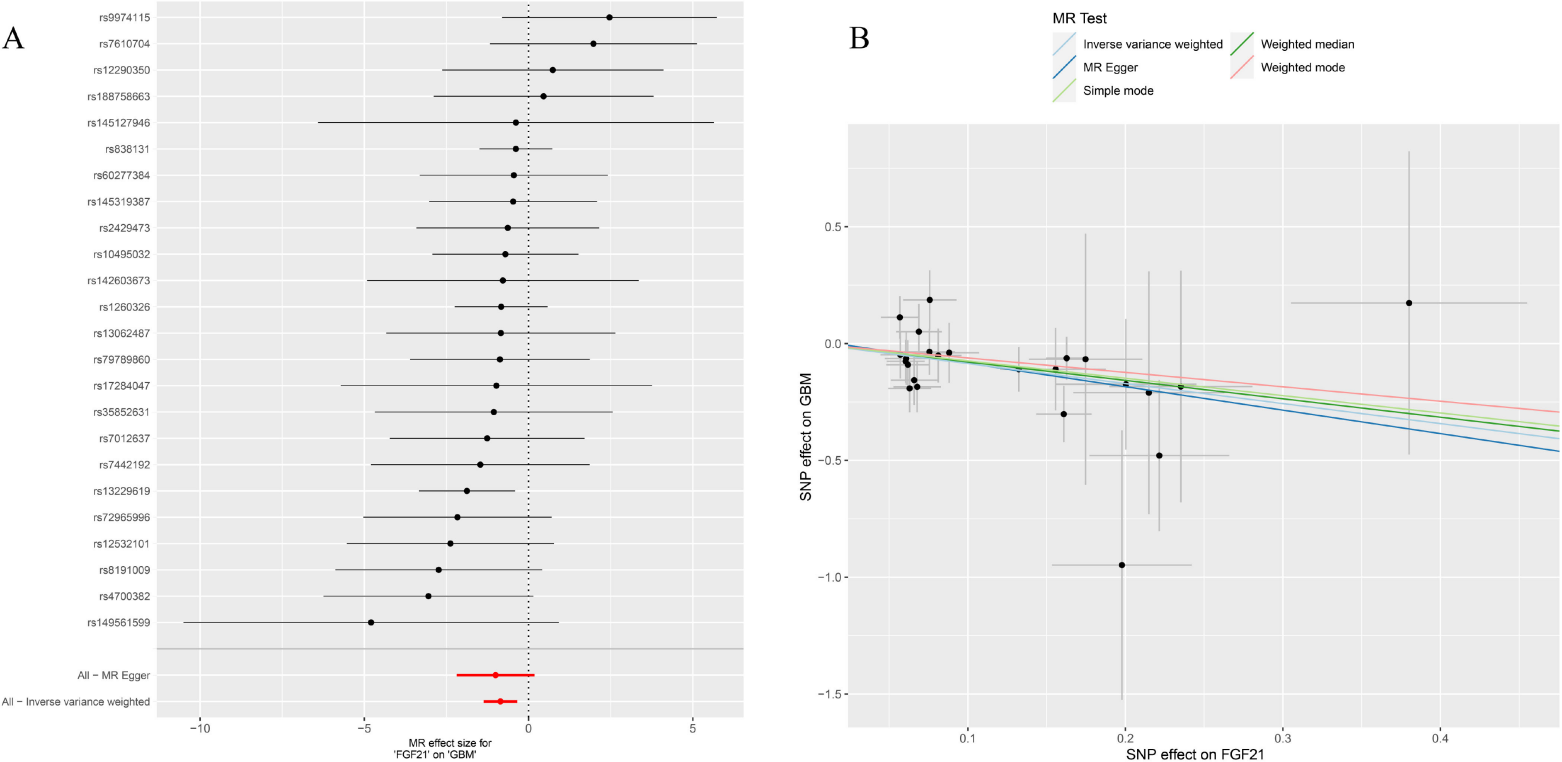
**(A)** Forest plot to visualize causal effect of each single FGF21 SNP on GBM; **(B)** the scatter plots to visualize causal relationship between FGF21 and GBM from different methods. SNP, single nucleotide polymorphism; MR, mendelian randomization; GBM, glioblastoma; FGF21, fibroblast growth factor 21.

After removing palindromic and ambiguous SNP, we extracted 24 genome-wide significant SNPs as instrumental variables. The F-statistics of the instrumental variables of FGF21 ranged from 19.6 to 156.6 (**Supplementary Table S4**). Based on the IVW method, FGF21 is significantly negatively correlated with GBM (OR = 0.42, 95% CI:0.25-0.71, P=0.001; **Figures 2**, **5A**). The other two **Supplementary Methods**, weighted median (OR =0.45, 95% CI:0.22-0.96, P=0.038) and BWMR (OR =0.44, 95% CI:0.26-0.73, P=0.0018), showed results consistent with the IVW method (**Table 1**, **Figure 5B**).

**Table 1:** The result of mediation analysis.

Total effect (βall)	Effect of PC16 on FGF21 (β1)	Effect of FGF21 on GBM (β2)	Mediation effect (β12)	Direct effect (βdirect)	Proportion mediated (%)
0.542	-0.062	-0.857	0.053	0.489	9.78

PC16, phosphatidylcholine 16:0_22:6; GBM, glioblastoma; FGF21, fibroblast growth factor 21.

### Proportion of the association between PC16 and GBM mediated by FGF21

We analyzed the mediating effect of FGF21 in the causal relationship between PC16 and GBM. We found that PC16 is associated with a decrease in FGF21, and a decrease in FGF21 is associated with an increased risk of GBM. This mediating effect accounts for 9.78% of the total causal effect (**Table 1**).

### Sensitive analysis

Several sensitivity analysis methods were used in this study to detect possible pleiotropy. The results of Cochran’s Q test showed no evidence of heterogeneity or asymmetry among these SNPs. In our study, the tests of MR-Egger and MR-PRESSO showed no horizontal pleiotropy (**Table 2**). Leave-one-out analysis was used to validate the influence of each SNP on the overall causal estimate. After removing each SNP, a systemic MR analysis was conducted again on the remaining SNPs, and the results remained consistent, indicating that all SNP estimations contribute significantly to the causal association. This also indicates that no dominant SNPs exist for PC16 with GBM, PC16 with FGF21, and FGF21 with GBM, and previous MR results are valid (**Supplementary Figures S1**–**S3**).

**Table 2:** Sensitive analysis results.

Exposure	Method	Heterogeneity		Pleiotropy		
		Q	P Value	MR-Egger Regression		MR-Presso
				Egger Intercept	P Value	Global Test P Value
**PC16 on GBM**	IVW	22.19	0.45	-0.058	0.37	0.45
	MR Egger	21.34	0.44			
**PC16 on FGF21**	IVW	18.23	0.69	0.008	0.25	0.70
	MR Egger	16.85	0.72			
**FGF21 on PC16**	IVW	18.22	0.75	0.016	0.79	0.76
	MR Egger	18.15	0.70			

PC16, phosphatidylcholine 16:0_22:6; GBM, glioblastoma; FGF21, fibroblast growth factor 21; IVW, inverse variance weighted; MR-Egger, Mendelian randomization-Egger; MR-Presso, Mendelian randomization-Pleiotropy RESidual sum and outlier.

## Discussion

This study employs the statistical result data from large-scale GWAS to analyze the causal relationship between PC16 and GBM, using a two-step Mendelian Randomization (MR) approach to assess the mediating effect of FGF21. The results suggest that PC16 is a risk factor for GBM, which can be mitigated through the mediation of FGF21 expression, thereby increasing the risk of GBM. MR-Egger and MR-PRESSO test analyses indicate that the MR findings are not affected by pleiotropy, and no dominant SNPs were present.

Phosphatidylcholine is not only a major component of cellular biomembranes but also plays a significant role in cellular signaling pathways. To accommodate the increased proliferation rate of cancer cells, an activation of lipid biosynthesis is observed to provide sufficient fatty acids for new membrane generation (22). Previous *in vivo* and *in vitro* MRS studies have shown that higher-grade gliomas exhibit elevated total choline levels compared to lower-grade gliomas (23–25), with other various tumors also demonstrating increased cellular total choline metabolites, including choline, phosphocholine, and phosphatidylcholine (10, 11). However, the potential causal relationship and mechanisms of action between PC16 and GBM have not been studied. This study establishes a causal relationship between the two.

FGF21, a hormone secreted by the liver, regulates simple sugar intake via signal transduction through the FGF21 receptor in the paraventricular nucleus of the hypothalamus, and is associated with a reduction in dopamine neurotransmission in the nucleus accumbens. FGF21 acts as a downstream metabolic target of oncogenes or as a corrective metabolic suppressor in tumor lesions (26, 27), with its overexpression antagonizing the development of liver and pancreatic cancers (28, 29). Furthermore, FGF21 inhibits pro-tumorigenic factors, including ER stress, ROS stress, and mitochondrial dysfunction (30–32). However, in GBM, upregulation of FGF21 gene expression promotes glioma cell protrusion elongation (33). Additionally, FGF21 may be inactivated by cleavage by Fibroblast Activation Protein (FAP), affecting GBM cell metabolic regulation and tumor progression (34, 35), This aligns with our findings that FGF21 may mediate the causal relationship between PC16 and GBM as an intermediary factor.

This study exhibits several strengths. To the best of our knowledge, this is the first MR study to investigate the causal relationship between PC16 and GBM, incorporating FGF21 to analyze the mediation proportion in this causal relationship. We minimized confounding factors and reverse causality to avoid potential biases inherent in observational studies. Alternative methods like MR-Egger, weighted median, and BWMR were used, and results were validated through sensitivity analysis, providing robust outcomes.

This study also has certain limitations. Firstly, although the data used encompass a large sample size, they are sourced from European ancestry populations. The expression of PC16, incidence of GBM, and expression of FGF21 vary among different populations. Whether the study results can be generalized to other populations requires further research using relevant GWAS data from diverse populations. Secondly, while the study employed an MR design and strived to rule out known confounders, the results may still be influenced by unconsidered potential confounders. Finally, while MR is a theoretical method for analyzing causal relationships, further laboratory research is needed to explore the specific biological connections between PC16 and GBM.

## Conclusion

In conclusion, this study confirmed the causal relationship between PC16 and GBM through MR analysis and identified that FGF21 plays a vital mediating role in this causal relationship.

## Data Availability

The original contributions presented in the study are included in the article/**Supplementary Material**. Further inquiries can be directed to the corresponding authors.

## Supplementary material

Supplementary Figure 1 the leave-one-out plots of the sensitivity analysis for PC16 on GBM. PC16, phosphatidylcholine 16:0_22:6; GBM, glioblastoma; MR, Mendelian randomization analysis.

Supplementary Figure 2 the leave-one-out plots of the sensitivity analysis for PC16 on FGF21. PC16, phosphatidylcholine 16:0_22:6; FGF21, fibroblast growth factor 21; MR, Mendelian randomization analysis.

Supplementary Figure 3 the leave-one-out plots of the sensitivity analysis for FGF21 on GBM. FGF21, fibroblast growth factor 21; GBM, glioblastoma; MR, Mendelian randomization analysis.
