# Supplementary figures and images for: Role of FGF21 in mediating the effect of phosphatidylcholine on GBM
Source: Front Oncol. 2024 Sep 2;14:1428025. doi: 10.3389/fonc.2024.1428025 (PMC11402610; doi:10.3389/fonc.2024.1428025)

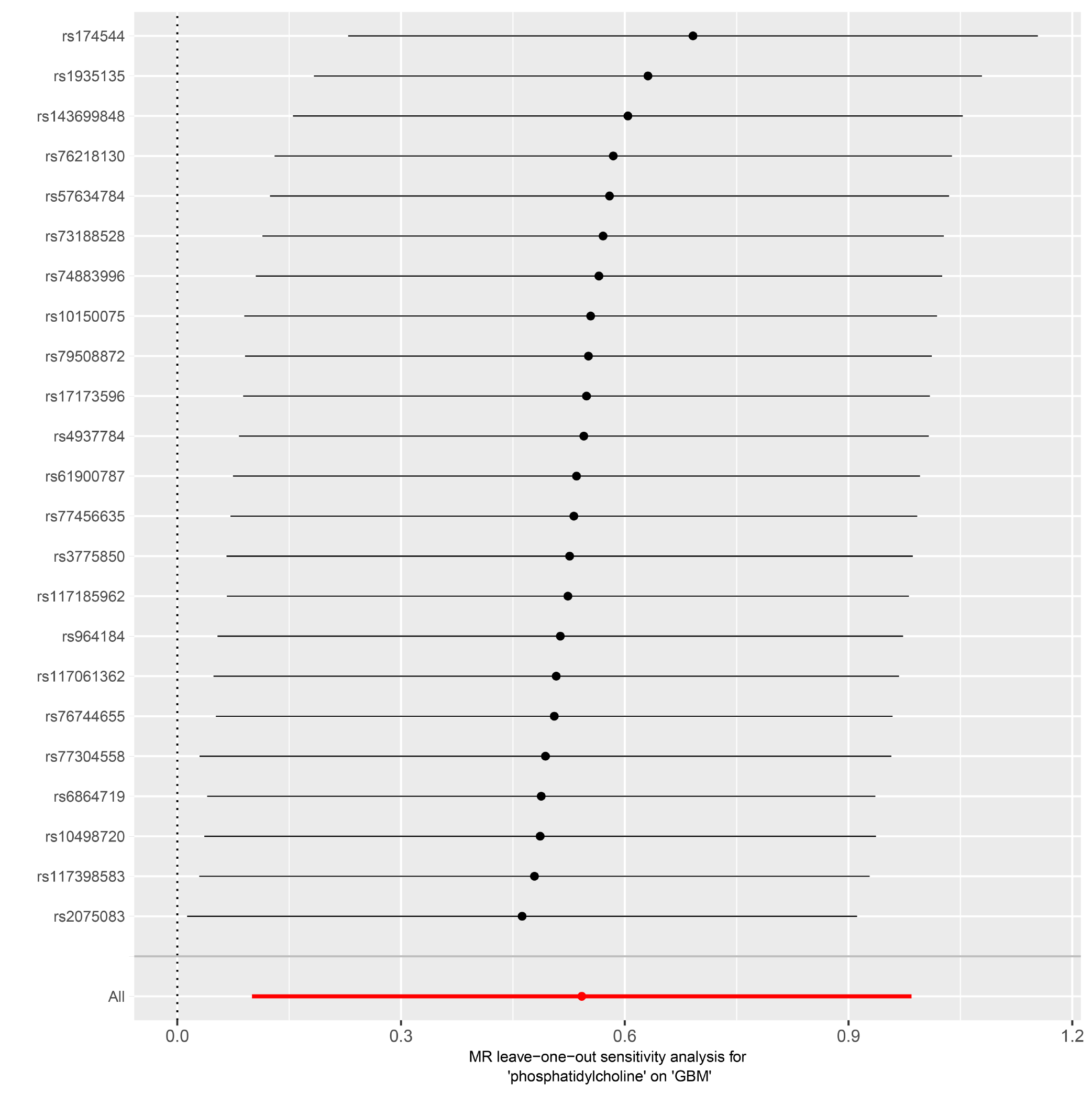

Supplement: Supplementary Figure 1 — the leave-one-out plots of the sensitivity analysis for PC16 on GBM. PC16, phosphatidylcholine 16:0_22:6; GBM, glioblastoma; MR, Mendelian randomization analysis. [file Image1.tif]

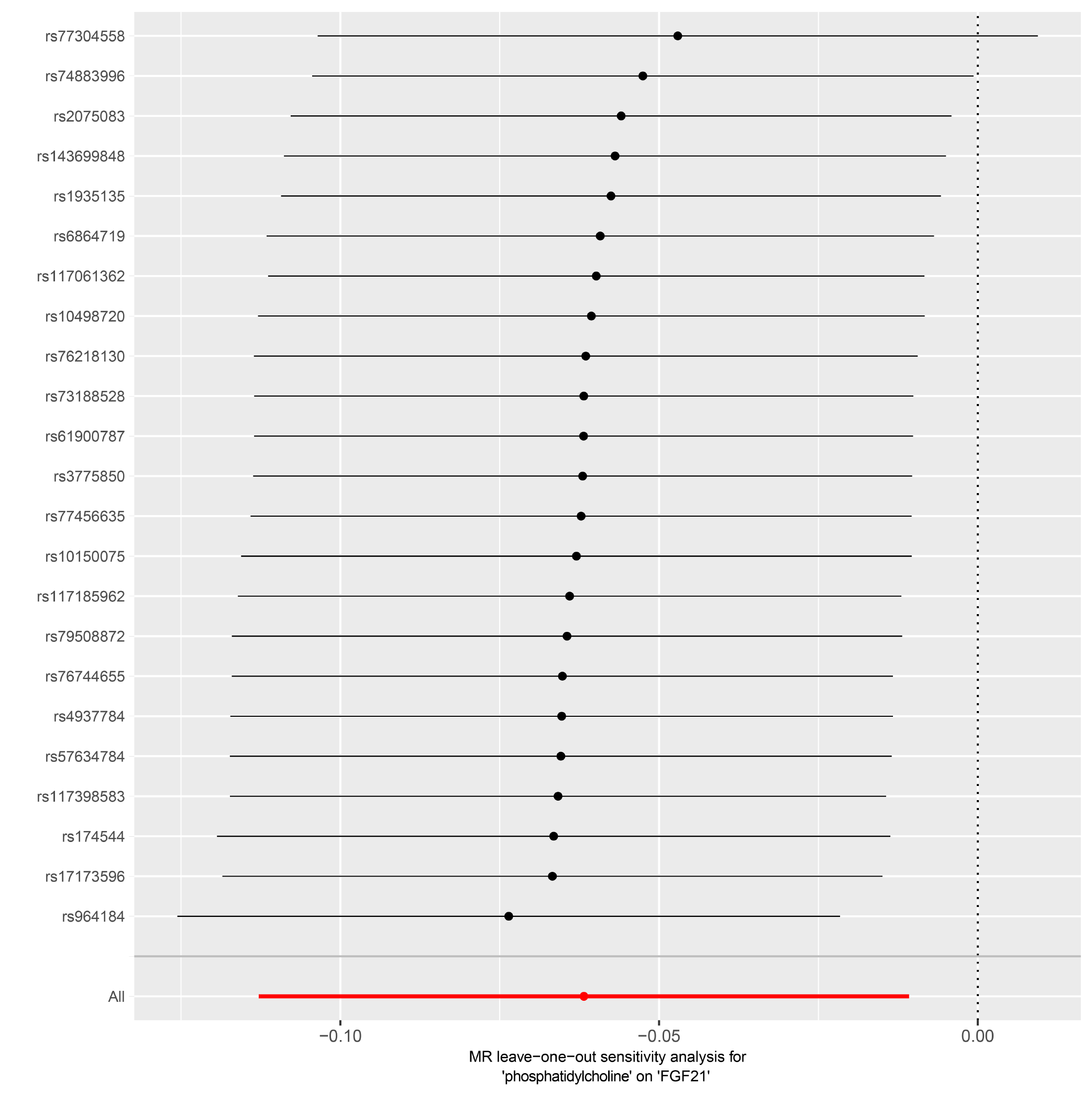

Supplement: Supplementary Figure 2 — the leave-one-out plots of the sensitivity analysis for PC16 on FGF21. PC16, phosphatidylcholine 16:0_22:6; FGF21, fibroblast growth factor 21; MR, Mendelian randomization analysis. [file Image2.tif]

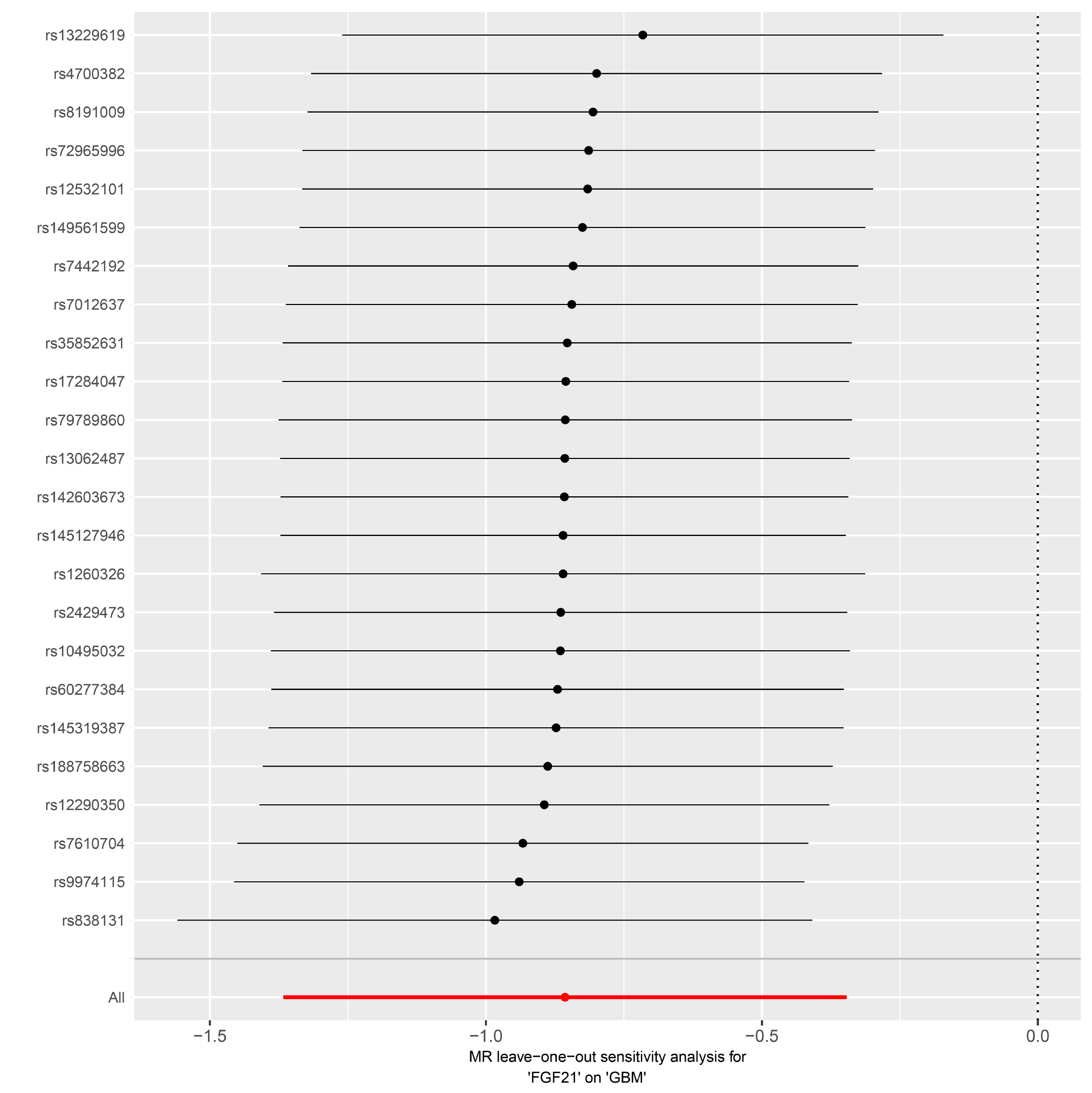

Supplement: Supplementary Figure 3 — the leave-one-out plots of the sensitivity analysis for FGF21 on GBM. FGF21, fibroblast growth factor 21; GBM, glioblastoma; MR, Mendelian randomization analysis. [file Image3.tif]
